# Supplementary figures and images for: High Transcriptional Activity and Diverse Functional Repertoires of Hundreds of Giant Viruses in a Coastal Marine System
Source: mSystems. 2021 Jul 13;6(4):e00293-21. doi: 10.1128/mSystems.00293-21 (PMC8407384; doi:10.1128/mSystems.00293-21)

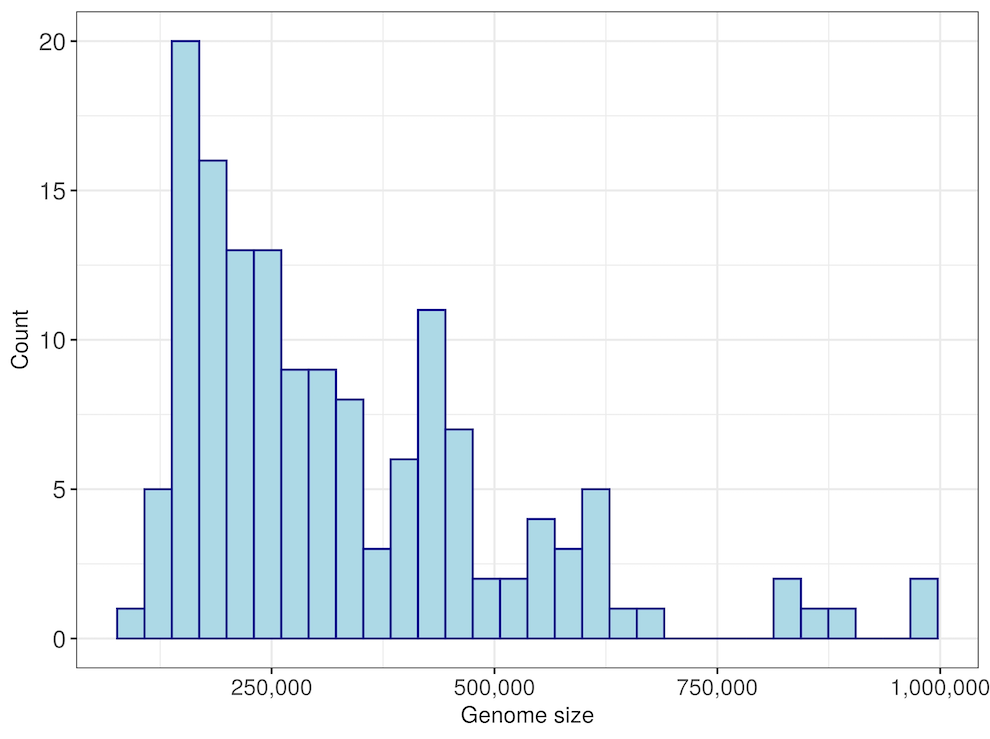

Supplement: FIG S1 [file msystems.00293-21-sf001.tif]

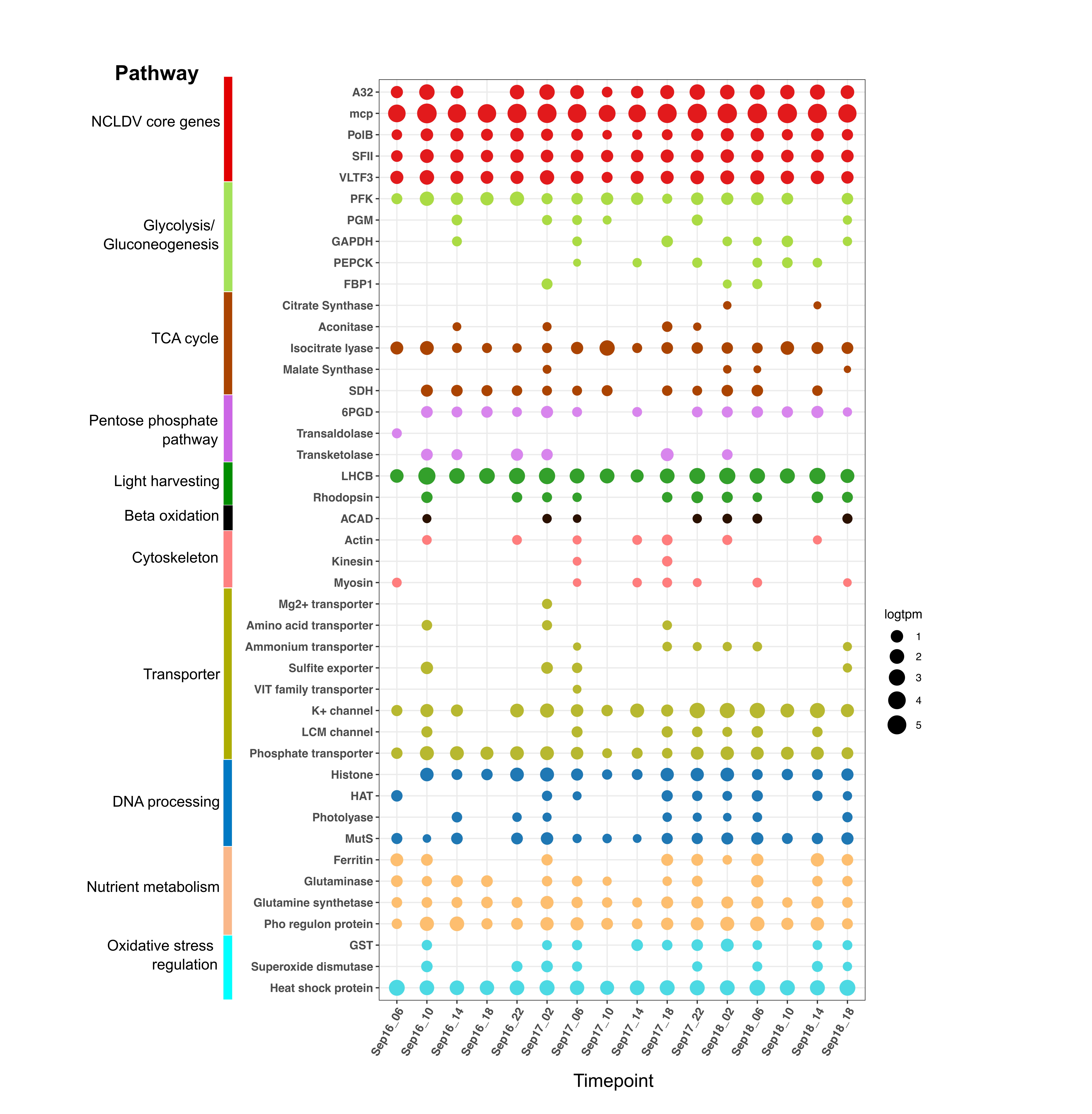

Supplement: FIG S2 [file msystems.00293-21-sf002.tif]
